# Concatenation Analyses in the Presence of Incomplete Lineage Sorting

**DOI:** 10.1371/currents.tol.8d41ac0f13d1abedf4c4a59f5d17b1f7

**Published:** 2015-05-22

**Authors:** Tandy Warnow

**Affiliations:** Department of Computer Science, University of Illinois at Urbana-Champaign. Urbana, Illinois, USA

## Abstract

Incomplete lineage sorting (ILS), modelled by the multi-species coalescent, is a process that results in a gene tree being different from the species tree. Because ILS is expected to occur for at least some loci within genome-scale analyses, the evaluation of species tree estimation methods in the presence of ILS is of great interest. Performance on simulated and biological data have suggested that concatenation analyses can result in the wrong tree with high support under some conditions, and a recent theoretical result by Roch and Steel proved that concatenation using unpartitioned maximum likelihood analysis can be statistically inconsistent in the presence of ILS. In this study, we survey the major species tree estimation methods, including the newly proposed “statistical binning” methods, and discuss their theoretical properties. We also note that there are two interpretations of the term “statistical consistency”, and discuss the theoretical results proven under both interpretations.

## Introduction

Estimating species trees from multiple loci is commonly performed using concatenation methods, in which multiple sequence alignments from different genomic regions are concatenated into one large supermatrix, and then a tree is estimated on the supermatrix. Yet, incomplete lineage sorting (ILS) (modelled by the multi-species coalescent model) can result in different loci having topologically different phylogenies, with high probability of gene tree incongruence when the effective population size is large and the time between speciation events is small 3. Most importantly, Roch and Steel 1 recently proved that using unpartitioned maximum likelihood to estimate a species tree on a concatenated alignment from different loci can converge to a tree other than the species tree as the number of loci increases, even if the sequence length per locus is allowed to increase; in other words, unpartitioned maximum likelihood can be statistically inconsistent (and even positively misleading, a stronger statement) under the multi-species coalescent model. Furthermore, simulation studies have shown that species trees estimated using concatenation can result in incorrect trees with high support 2. Thus, both theory and empirical studies show that the use of concatenation to estimate a species tree from multiple loci can lead to incorrect phylogenetic estimations.

Because concatenation can result in incorrect trees, coalescent-based "species tree methods" have been developed that are statistically consistent under the multi-species coalescent model. However, there are two meanings for "statistical consistency under the multi-species coalescent model", and so it is important to distinguish between them.

- Statistical consistency under the multi-species coalescent model - weak version. The most common use of the term asserts that the tree estimated by the species tree method will converge in probability to the true species tree as the number of sites per locus and the number of loci both increase 3.
- Statistical consistency under the multi-species coalescent model - strong version. The other use of the term asserts that the estimated tree will converge in probability to the true species tree as the number of loci increases, but limits the sequence length per locus (perhaps to a constant number of sites). The first use of the term is clearly the weaker condition, since it makes stronger assumptions.

Thus, what Roch and Steel proved 1 is that unpartitioned maximum likelihood is statistically inconsistent in both senses. However, their proof does not extend to fully partitioned maximum likelihood analyses, which allow the numeric model parameters to change between the different loci in the concatenated alignment. Indeed, it is not yet established whether unpartitioned maximum likelihood analyses are inconsistent or consistent under either of these interpretations of the meaning of statistical consistency.

On the other hand, many species tree estimation methods have been developed that are provably statistically consistent in the first (i.e., weaker) sense of the term, using a variety of algorithmic approaches. For example, some methods (e.g., *BEAST 20) co-estimate gene trees and species trees) and others (e.g., SVDquartets 15) estimate the tree using a single site from each locus within a set of unlinked loci; however, most of the commonly used methods estimate the species tree by combining estimated gene trees. These "summary methods" (such as MP-EST 12 and ASTRAL 4) are statistically consistent under the multi-species coalescent model in the first (i.e., weaker) sense of the term, and are increasingly popular due to their relative speed and ease of use.

Only a few coalescent-based species tree estimation methods have been proven to be statistically consistent in the second (i.e., stronger) sense of the term, which establishes that the species tree estimated by the method converges to the true species tree as the number of loci is allowed to increase, even when the sequence length per locus is bounded 7 ^,^ 16 ^,^ 18 ^,^ 19. However, it is not known whether the commonly used summary methods such as MP-EST are statistically consistent under the second sense of the term.

Simulation studies evaluating the relative performance of species tree estimation methods in comparison to concatenation analysis have had mixed results (e.g., sometimes concatenation is more accurate, sometimes coalescent-based methods are more accurate, and sometimes the differences are not statistically significant 4 ^,^ 5 ^,^ 6 ^,^ 8 ^,^ 9 ^,^ 10 ^,^ 11). In addition, coalescent-based summary methods have been shown to have reduced accuracy in the presence of substantial gene tree estimation error 4 ^,^ 5 ^,^ 6 ^,^ 8 ^,^ 10 ^,^ 11. Furthermore, the proofs of statistical consistency for standard coalescent-based summary methods (such as MP-EST and ASTRAL) have assumed that all the input gene trees are estimated without any error 7. Since estimated gene trees are often imperfect, the assumption of 100% accurate gene trees is unlikely to be biologically realistic 8.

## Statistical Binning

In two recent papers 10 ^,^ 11, we presented techniques designed to improve coalescent-based species tree estimation by improving gene tree estimation when estimated gene trees have insufficient accuracy as a result of limited phylogenetic signal (e.g., because of low rates of evolution or sequence lengths that are too short). These two techniques partition the loci into sets, so that each set should contain loci that are deemed likely to have a common evolutionary tree. The first of these techniques 11 is called “unweighted statistical binning” and the second 10 is called “weighted statistical binning”; the use of weighting in statistical binning addresses a weakness we identified in unweighted statistical binning. Each type of statistical binning uses a fully partitioned maximum likelihood analysis to estimate a tree on each bin, and then these "supergene" trees are given to a summary method (such as MP-EST) to compute a species tree. Hence, statistical binning is a combination of concatenation and coalescent-based species tree estimation.

Statistical binning has the following steps:

1. Compute gene trees with bootstrapping on each locus, using maximum likelihood.
2. Use the bootstrap support on the edges of each gene tree to determine for each pair of genes whether they are likely to have a common gene tree topology, and build an "incompatibility graph" to represent this information (so that each node represents a gene, and two genes are connected by an edge if the topological differences between their gene trees are considered statistically significant according to the test).
3. Partition the vertices of the graph into sets of approximately the same size, so that no two vertices in any set are adjacent; these are called the "bins".
4. For each bin, concatenate the alignments in the bin, and compute a fully partitioned maximum likelihood tree on the bin; these are called the "supergene trees". *If performing a weighted statistical binning, then replicate the supergene tree by the number of genes in its bin. If performing an unweighted statistical binning, then do not replicate the supergene trees.*
5. Apply the preferred summary method (e.g., ASTRAL or MP-EST) to the set of supergene trees to compute the species tree.

Note that the only difference between weighted and unweighted statistical binning is that weighted statistical binning replicates the supergene trees by the number of genes in the bin, and unweighted statistical binning does not do this. This difference is essential to the theoretical properties of the two methods. As proven in 10, pipelines based on weighted statistical binning followed by summary methods such as MP-EST or ASTRAL (that will converge to the true species tree as the number of true gene trees increases) are statistically consistent using the first definition, which allows the number of sites per locus as well as the number of loci to increase. However, unweighted statistical binning - even if followed by MP-EST or ASTRAL - is not statistically consistent under the first definition!

As shown in 10, MP-EST and ASTRAL used with weighted statistical binning were typically more accurate than when used without weighted statistical binning with respect to the estimation of species tree topologies and branch lengths, on datasets simulated under the multi-species coalescent model. However, there are some cases where using weighted statistical binning reduced accuracy, but these are limited to small numbers of taxa with very high levels of ILS.

Thus, statistical binning used with a coalescent-based summary method provides a blend of concatenation and coalescent-based methods: supergene trees are computed on concatenated alignments using fully partitioned maximum likelihood analyses, and then a coalescent-based summary method (such as MP-EST) is applied to these supergene trees to estimate a species tree. However, importantly, all maximum likelihood analyses used in statistical binning are fully partitioned, and this is critically important to the statistical properties that can be proven about these methods.

Liu et al. 17 argue against the use of statistical binning, saying "We show that approaches such as binning, designed to augment the signal in species tree analyses, can distort the distribution of gene trees and are inconsistent". The claim that they show phylogenomic pipelines using naive binning 21 (not the same as statistical binning) are inconsistent is invalid, since they argue through the use of simulations (their own, and by reference to other simulations). However, statistical consistency or inconsistency is a property of behavior as the amount of data goes to infinity -- and simulations are limited to finite data. Therefore, simulations by definition cannot prove statistical consistency or inconsistency. *Hence, their paper does not establish inconsistency for naive binning, nor for any kind of binning approach.*

Although their simulation study only examined naive binning 21, Liu et al. also expressed concerns about the potential for statistical binning to have a deleterious effect on species tree estimation. Therefore, their study does raise the following question: *Are statistical binning pipelines statistically consistent under the multi-species coalescent model?*

We can definitively answer this question with respect to the first meaning of statistical consistency: as shown in 10, phylogenomic pipelines that use weighted statistical binning followed by coalescent-based summary methods such as MP-EST will converge in probability to the true species tree as the number of loci and sites per locus both increase. We sketch the proof, to illustrate how the different aspects of the algorithm design are used to ensure statistical consistency. First, 10 showed that as the sequence length per locus increases, estimated gene trees converge to the true gene tree, and their bootstrap support values also converge to 100%; hence, the binning procedure produces bins that contain genes with the same gene tree topology with probability converging to 1. Recall that in a weighted statistical binning analysis, the loci that are placed in the same bin are then analyzed under a *fully partitioned* maximum likelihood analysis. Under the assumption that all the loci in the same bin have evolved down the same tree, a fully partitioned maximum likelihood analysis will converge to the tree associated to the bin as the length of the sequences for each locus increased. Then, in a weighted statistical binning analysis, this “supergene” tree would be replicated as many times as the number of loci in its bin. Finally, as the number of loci increases, the distribution of the supergene trees will converge to the true gene tree distribution defined by the species tree. Therefore, if the supergene trees are analyzed using a summary method (e.g., MP-EST or ASTRAL) that is statistically consistent given true gene trees, the pipeline described would be statistically consistent (in the first sense) under the multi-species coalescent model.

However, as shown in 10, pipelines using unweighted statistical binning (which does not replicate the supergene trees) are very different. In particular, as the number of genes increases, the estimated distribution of supergene trees will converge to the flat distribution in which every possible gene tree topology appears exactly once. Hence, no matter how these supergene trees are combined, the true species tree cannot be estimated with high probability. Therefore, pipelines that use unweighted statistical binning are not consistent even in the weak sense.

But what about the second interpretation of statistical consistency, where the number of sites per locus is fixed, but the number of loci increases? Are pipelines that use weighted statistical binning, followed by a summary method such as MP-EST or ASTRAL, statistically consistent under this second meaning (i.e., the strong sense of statistical consistency)? Does the Roch and Steel result help shed any light on this issue? Before we can answer this, we need to understand the difference between unpartitioned and fully partitioned maximum likelihood analyses.

## PARTITIONED AND UNPARTITIONED MAXIMUM LIKELIHOOD ANALYSES

Suppose we are given multiple sequence alignments for *p* different loci, and we wish to compute a maximum likelihood tree on the concatenated alignment under the Jukes-Cantor site evolution model, where a Jukes-Cantor model tree consists of a rooted binary tree T and numeric model parameters (the branch lengths of the tree). In an unpartitioned Jukes-Cantor maximum likelihood analysis we assume all the sites within the alignment evolve down a single Jukes-Cantor model tree, and we seek the tree and its numeric model parameters that is most likely to have generated the observed data.

In a fully partitioned Jukes-Cantor maximum likelihood analysis, we no longer assume that all the sites evolve down a single Jukes-Cantor model tree; instead, we assume that the different parts of the concatenated alignment each evolves down its own model tree, and the only constraint we make is that the model tree for the different parts share the same tree topology. Hence, we allow the numeric model parameters (i.e., branch lengths) to differ between the different loci. Therefore, the result of a fully partitioned Jukes-Cantor maximum likelihood analysis on a concatenated alignment from *p* loci is a set of *p* (potentially different) Jukes-Cantor model trees, each sharing the same tree topology. Equivalently, the result is a single tree topology T, but also *p* branch lengths for each branch in T.

We will show that fully partitioned maximum likelihood analyses and unpartitioned maximum likelihood analyses have very different theoretical properties. First, consider how maximum likelihood evaluates a sequence alignment where the sequences for the different species are all identical (i.e., an input of 100 sequences each identical to AACATAG). It is easy to see that all tree topologies are equally good under Jukes-Cantor maximum likelihood, and so cannot be distinguished under the maximum likelihood criterion. We will refer to alignments of this type as "invariant alignments" and loci that have invariant alignments as "invariant loci". Now consider a concatenated alignment based on *p* loci, where all the loci but one are invariant. Under a fully partitioned Jukes-Cantor maximum likelihood analysis of such a dataset, because the *p*-1 invariant multiple sequence alignments fit every tree topology equally well, they do not impact the fully partitioned Jukes-Cantor maximum likelihood analysis of the concatenated alignment, and the result is identical to the Jukes-Cantor maximum likelihood analysis of the single variable multiple sequence alignment (see 10 for details) . However, as Roch and Steel showed (see below), unpartitioned maximum likelihood analyses converge (as the number of loci increases) to a maximum parsimony analysis of the single variable multiple sequence alignment. Thus, unpartitioned and fully partitioned maximum likelihood analyses behave very differently, and theoretical results (positive or negative) about one do not imply the same results for the other.

## Roch and Steel's proof that concatenation is statistically inconsistent under the multi-species model

Roch and Steel 1 prove that unpartitioned maximum likelihood is statistically inconsistent under the multi-species coalescent model, under the assumption that the gene sequence evolution model is the r-state symmetric model (i.e., models such as Jukes-Cantor, in which substitutions between every pair of distinct states are equiprobable). They establish this proof by showing that for some model species tree with very high levels of ILS and very low rates of evolution (in which all loci have the same sequence length), with high probability, nearly all loci will be invariant (i.e., their sequence alignments will have no changes on them). They then show that under the r-state symmetric model, unpartitioned maximum likelihood will converge to maximum parsimony as the number of loci increases (see Appendix). Roch and Steel then argue that under these conditions, as the number of loci increases, maximum parsimony will converge to a tree that is different from the species tree. In other words, unpartitioned maximum likelihood on the concatenated alignment will be positively misleading because maximum likelihood will be identical to maximum parsimony under some conditions (for large enough numbers of loci), and maximum parsimony will be positively misleading under the multi-species coalescent model.

Consider an input where the first *p*-1 loci have invariant multiple sequence alignments, and the *p* ^th^ locus has a variable sequence alignment. By the argument provided by Roch and Steel, as *p*→∞, an unpartitioned maximum likelihood analysis will converge to a maximum parsimony analysis of the concatenated alignment. Since *p*-1 of these loci are invariant, the unpartitioned Jukes-Cantor maximum likelihood analysis of the concatenated alignment converges to the maximum parsimony analysis of the *p* ^th^ locus.

For the same data, but under a fully partitioned maximum likelihood analysis, the first *p*-1 loci have no impact on the GTR maximum likelihood analysis, and so the result is identical to Jukes-Cantor maximum likelihood on the *p* ^th^ locus. Thus, for all values of p, the fully partitioned maximum likelihood analysis will always be the Jukes-Cantor maximum likelihood of the *p* ^th^ locus. Thus, as *p*→∞, the unpartitioned maximum likelihood analysis will converge to the maximum parsimony analysis of the *p* ^th^ locus but the fully partitioned maximum likelihood analysis will be the maximum likelihood analysis of the *p* ^th^ locus. Since Jukes-Cantor maximum likelihood and maximum parsimony are not identical methods (i.e, they can return different trees on the same dataset), this shows that fully partitioned and unpartitioned maximum likelihood analyses are different methods, and can return different trees.

To summarize, the theorem by Roch and Steel established that an *unpartitioned* maximum likelihood analysis can be inconsistent under the multi-species coalescent model, but their result *does not apply to a fully partitioned maximum likelihood analysis.* Most importantly, their proof uses the fact that the maximum likelihood analysis is unpartitioned to show that as the number of loci increases and nearly all the loci are invariant, the unpartitioned maximum likelihood analysis converges to maximum parsimony. This statement is explicitly not true for fully partitioned analyses, which are unaffected by invariant loci. Hence, their entire argument is restricted to unpartitioned maximum likelihood, and their proof does not apply to a fully partitioned maximum likelihood analysis.

## Summary and Discussion

This article has focused on what has been established theoretically for some standard methods for estimating species trees (i.e., concatenation using maximum likelihood and summary methods such as MP-EST and ASTRAL), as well as for the newer approach of weighted statistical binning, followed by a summary method. Because the term "statistical consistency" has been used in two different ways in the literature, we have summarized what is known under each meaning: the weaker sense where both parameters (sequence length per locus and number of loci) increase, and the stronger sense where only the number of loci increases but the sequence length per locus is bounded, perhaps by a constant. We have also clarified that Roch and Steel's theorem about concatenation using maximum likelihood being statistically inconsistent is restricted to unpartitioned maximum likelihood.

So, what do we know about statistical consistency (for either sense) of standard techniques for estimating species trees in the presence of ILS? Methods that have been proven to be statistically consistent in the first sense include the standard summary methods (e.g., MP-EST, ASTRAL, the population tree from BUCKy 22, etc.), methods that estimate trees directly from alignments, such as *BEAST and SVDquartets, and also weighted statistical binning, paired with standard summary methods. On the negative side, Roch and Steel's theorem establishes that unpartitioned maximum likelihood can be statistically inconsistent. A separate argument shows that unweighted statistical binning is inconsistent in this first sense.

However, even for this weak sense of statistical consistency (where both the number of sites per locus and number of loci increase), the statistical consistency of many methods is still unknown. In particular, Roch and Steel's theorem does not establish statistical inconsistency for fully partitioned maximum likelihood. Because the loci can have different tree topologies under the multi-species coalescent model, it seems likely that maximum likelihood analyses, even if fully partitioned, will be found to be inconsistent. However, the proof for an unpartitioned analysis being inconsistent provided by Roch and Steel in 1 depends on using an unpartitioned analysis, and so establishing the inconsistency (or consistency, as the case may be) of fully partitioned maximum likelihood analysis requires a different mathematical argument.

In terms of the second definition (and stronger sense) of statistical consistency, where only the number of loci increase but the number of sites per locus is bounded, very little has been established. In fact, the only established results are that unpartitioned maximum likelihood and unweighted statistical binning are both inconsistent. Finally, we do not know whether any of the standard summary methods, fully partitioned maximum likelihood analyses, or weighted statistical binning, are statistically consistent under this second definition.

In fact, to try to prove a method is statistically consistent or inconsistent under the second definition is very difficult; the only methods that have been proven to be statistically consistent under this definition (which constrains the number of sites per locus to be bounded) are explicitly designed to have this property, but all (to our knowledge) require some additional constraints (e.g., the same constant rate of evolution across all loci 19 , or a strict molecular clock 7). Proofs of inconsistency under the second definition have been established, but again only for unpartitioned maximum likelihood (proven by Roch and Steel) and unweighted statistical binning. Attempts to prove statistical consistency or inconsistency under this second definition have so far failed for any of the standard methods.

In other words, the major phylogenomic estimation methods in common use that are designed to address incomplete lineage sorting -- summary methods such as MP-EST, ASTRAL, and the population tree from BUCKy, and co-estimation methods such as *BEAST -- have been proven to be statistically consistent only in the first sense, where both the number of loci and number of sites increase. None of them have been proven to be consistent in the second sense, where the sequence lengths per locus are bounded. Similarly, the weighted statistical binning method is statistically consistent in this first sense, but it is not known if it is statistically consistent in the second sense.

It is also clear that Roch and Steel's theorem (as well as the observations provided by their proof) is limited to unpartitioned maximum likelihood. Therefore, it cannot help us understand the theoretical properties of fully partitioned maximum likelihood, and hence also cannot help us understand the theoretical properties of methods that use fully partitioned maximum likelihood (such as weighted statistical binning). Therefore, any attempt to establish the statistical consistency or inconsistency of these methods will need to use other mathematical arguments.

In other words, the established theory regarding species tree estimation methods is limited. Yet, performance in practice (i.e., on simulated and biological data) suggests that many methods (including concatenation) provide good accuracy under some model conditions. Unfortunately, as discussed in 7 ^,^ 8 ^,^ 10 ^,^ 13, most simulation studies have explored performance only on very small datasets (e.g., with at most ten species) and under unrealistic conditions. For example, 14 examined performance on model species tree with only 5 species and very high levels of ILS, where sequence evolution was under a strict molecular clock, and used 1000 sites per locus (so that the sequence lengths per locus are too large to avoid recombination events. Instead, to understand the relative performance of coalescent-based summary methods and concatenation, more extensive analyses based on biologically realistic conditions (and hence based on short sequences, or modeling sequence evolution with recombination within loci) are needed.

Finally, despite the interest in coalescent-based species tree estimation, the current methods are still in their infancy, and new methods will need to be developed in order to obtain highly accurate species trees under realistic conditions. While the focus of this article has been on the performance of concatenation analyses, and the implications for coalescent-based summary methods that use weighted statistical binning, alternative approaches have been developed that do not require the estimation of gene trees or supergene trees (e.g., 10 11 15 16 18 19 20). Given the theoretical and empirical challenges in producing accurate gene trees, these approaches may provide the best accuracy for genome-scale phylogenomic analysis.

## Statistical consistency (both senses) of standard species tree estimation techniques

**Statistical consistency of some standard methods:** We present the current status with respect to statistical consistency (of the first or second kind) of some standard phylogenomic estimation methods. The first column is for the first meaning of statistical consistency, which states that the species tree estimated by the method will converge to the true species tree as the number of loci and number of sites per locus both increase. The second column is for the second meaning, which states that the species tree estimated by the method will converge to the true species tree as the number of loci increases, even for bounded number of sites per locus. We also cite the paper in which the theoretical result is established.

	Consistency - first kind	Consistency - second kind
MP-EST	YES	UNKNOWN
ASTRAL	YES	UNKNOWN
Unpartitioned concatenated maximum likelihood	NO (1)	NO (1)
Fully partitioned maximum likelihood	UNKNOWN	UNKNOWN
Unweighted statistical binning followed by consistent summary method (e.g., ASTRAL)	NO (10)	NO (10)
Weighted statistical binning followed by consistent summary method (e.g., ASTRAL)	YES (10 )	UNKNOWN
*BEAST	YES	UNKNOWN

## Appendix 1: Quote from Roch and Steel's Paper

An outline of the proof of the main theorem is as follows: We show that the expected proportion of sites that are constant can be made arbitrary large with low rates of evolution (the lower bounds are formalized in Claim 4) and that the empirical frequencies of site patterns is concentrated around the expected values (Claim 2). When there are a large enough number of invariable sites, it can be shown that likelihood scores and parsimony scores converge to the same answer (formalized in Claim 1). Thus trees that have better parsimony score have better likelihood under these scenarios. Therefore, it suffices to show that parsimony is not statistically consistent under arbitrary low rates of evolution.

Sebastien Roch and Mike Steel, "Likelihood-based tree reconstruction on a concatenation of sequence datasets can be statistically inconsistent", Theoretical Population Biology 100 (2015): 56-62

## Competing Interests

The authors have declared that no competing interests exist.
